# SGR: an online genomic resource for the woodland strawberry

**DOI:** 10.1186/1471-2229-13-223

**Published:** 2013-12-23

**Authors:** Omar Darwish, Janet P Slovin, Chunying Kang, Courtney A Hollender, Aviva Geretz, Sam Houston, Zhongchi Liu, Nadim W Alkharouf

**Affiliations:** 1 Department of Computer and Information Sciences, Towson University, 7800 York Road, Towson, MD 21252, USA; 2 USDA/ARS Genetic Improvement of Fruits and Vegetables Laboratory, BARC-W 10300 Baltimore Ave, Beltsville, MD 20705, USA; 3 Department of Cell Biology and Molecular Genetics, University of Maryland, 0229 Biological Science Research Building, College Park, MD 20742, USA

**Keywords:** Strawberry, Transcriptome, RNA-seq, Database, gBrowse, Fruit, Flowers, Rosaceae

## Abstract

**Background:**

*Fragaria vesca*, a diploid strawberry species commonly known as the alpine or woodland strawberry, is a versatile experimental plant system and an emerging model for the Rosaceae family. An ancestral *F. vesca* genome contributed to the genome of the octoploid dessert strawberry (*F.* ×*ananassa*), and the extant genome exhibits synteny with other commercially important members of the Rosaceae family such as apple and peach. To provide a molecular description of floral organ and fruit development at the resolution of specific tissues and cell types, RNAs from flowers and early developmental stage fruit tissues of the inbred *F. vesca* line YW5AF7 were extracted and the resulting cDNA libraries sequenced using an Illumina HiSeq2000. To enable easy access as well as mining of this two-dimensional (stage and tissue) transcriptome dataset, a web-based database, the Strawberry Genomic Resource (SGR), was developed.

**Description:**

SGR is a web accessible database that contains sample description, sample statistics, gene annotation, and gene expression analysis. This information can be accessed publicly from a web-based interface at http://bioinformatics.towson.edu/strawberry/Default.aspx. The SGR website provides user friendly search and browse capabilities for all the data stored in the database. Users are able to search for genes using a gene ID or description or obtain differentially expressed genes by entering different comparison parameters. Search results can be downloaded in a tabular format compatible with Microsoft excel application. Aligned reads to individual genes and exon/intron structures are displayed using the genome browser, facilitating gene re-annotation by individual users.

**Conclusions:**

The SGR database was developed to facilitate dissemination and data mining of extensive floral and fruit transcriptome data in the woodland strawberry. It enables users to mine the data in different ways to study different pathways or biological processes during reproductive development.

## Background

The strawberry is well recognized throughout the world as a delicious and health promoting food. Strawberries are an important fruit crop in the United States, with an annual market value of over one billion dollars (The California Strawberry Commission, http://www.calstrawberry.com). The commercially grown dessert strawberry, *Fragaria* × *ananassa*, is an allo-octoploid hybrid of two octoploid species originating in the Western Hemisphere. The diploid *F. vesca*, thought to have contributed to the genome of *F.* ×*ananassa*, is commonly known as the woodland or alpine strawberry. *F. vesca* has a small sequenced genome (240 Mb), a small stature and short seed to seed cycle, and the ability to reproduce sexually and vegetatively, all of which have contributed to its usefulness as a reference plant for the genus [1]. In addition, *F. vesca* is transformable with *Agrobacterium tumefaciens*[2,3], so this small perennial is also an ideal candidate for functional genomic analyses within the Rosaceae family, which includes many important tree fruit crops such as apple, peach and cherry. Although there is a wide diversity of plant and fruit types within the Rosaceae, there remains sufficient synteny among strawberry, peach, and apple genomes [4] so that *F. vesca* can be considered an ideal system with which to study flower development, and to begin to understand the bases for the diverse fruit development within the family.

Due to the economic value of strawberry fruit, early molecular studies on fruit were concentrated on economically important processes such as flavor and aroma development, nutritional attributes, firmness, and ripening [5]. In contrast, little is known about the molecular regulation of strawberry floral organ and early fruit development. From an agricultural point of view, proper floral organ and gamete formation is essential for fruit development following fertilization. From a basic biological and evolutionary point of view, signaling between the sporophyte and the gametophytic cells within each sexual organ and between achene and receptacle is critical for proper seed maturation, fruit ripening, and seed dispersal. Next-generation sequencing (Illumina RNA Seq) was used to profile transcriptomes of early stage fruit development, with five fruit tissue types and five developmental stages from floral anthesis to enlarged fruits [6]. The ultimate goal is to allow scientists to investigate the molecular mechanisms underlying fruit development. The RNA-seq data from a total of 50 libraries (two replicates per tissue type) are currently available at the SGR, which will be updated as further data such as flower development transcriptomes become available. The extensive two dimensional (tissue and stage) digital data set on strawberry reproductive development can be mined by any researcher and serves as a valuable resource.

## Construction and content

The SGR database was designed, implemented, and hosted using Microsoft SQL Server 2008 R2 Enterprise Edition. Microsoft Visual Studio 2008 was used to design and implement the web pages, which were programmed using ASP.NET framework 2.35 with C# programming language. Both the SGR database and the website are hosted on the same web server located at Towson University in Baltimore, MD, USA. This server is running Microsoft Windows Server 2003 and Internet Information Services (IIS V6.0).

The SGR database stores descriptions of each of the replicated study samples, the number of reads of each sample, the quality filtration rates for the reads, the rates of alignment of reads to the genome, the rates of alignment of reads to genes, gene function information, gene ontology (GO) assignments, plant ontology (PO) assignments, and gene expression analyses using two different tools, DEGseq [7] and DESeq [8].

Gbrowse 2.0 [9] graphically displays the genome sequences with tracks showing predicted gene models for each of the samples and short reads from all of the study samples. The seven *F. vesca* pseudomolecules assembly file and a non-anchored scaffolds file were downloaded from the Genome Database for Rosaceae, GDR, (http://www.rosaceae.org/species/fragaria/fragaria_vesca/genome_v1.1) and merged together to be displayed representing the seven linkage groups of the genome. A GFF3 file of the GeneMark hybrid gene models (ftp://ftp.bioinfo.wsu.edu/species/Fragaria_vesca/Fvesca-genome.v1.1/genes/fvesca_v1.1_genemark_hybrid.gff3.gz) was downloaded and imported into MySQL server 5.1.67. All alignment output files were converted into a GBrowse acceptable format using samtools [10], thereby allowing them to be viewed as separate tracks. GBrowse and MySQL are hosted on a Linux server running Red Hat Enterprise Linux Server release 6.4.

## Utility and discussion

The SGR is the first web-accessible database for genome-scale transcriptomic analysis of early stage fruit development in woodland strawberry. The website gives public access to transcriptome data for the analysis of underlying molecular changes that accompany the morphological changes occurring during development in all sequenced tissues, as described in [6].

### RNA-sequencing analysis

**Figure 1:**
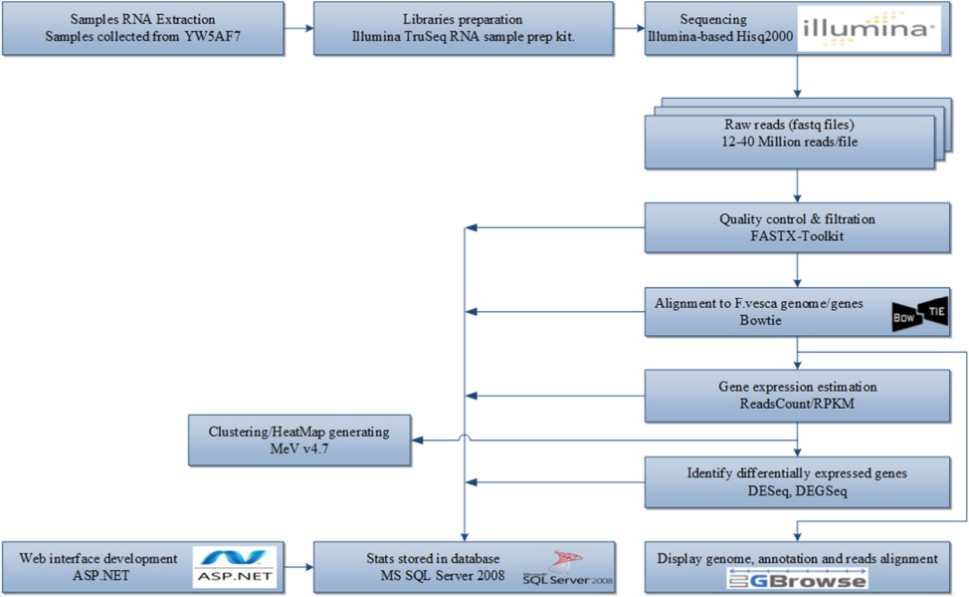
The bioinformatic pipeline used for SGR RNA-seq data analysis.

RNA-Seq is rapidly outcompeting microarrays for in-depth transcriptome studies. The Illumina Hiseq 2000 platform was used to obtain between 12 and 40 million 51 bp single end reads from each of 25 replicated libraries, for a total of ~70 Gb of sequenced data. The short reads were mapped to the *F. vesca* cultivar Hawaii 4 × 4 genome, which is the sequenced *F. vesca* cultivar [1]. After mapping, the number of reads hitting known genes in the genome were counted. The counts were then normalized to take into account the size of individual genes and the total sample size. We used the RPKM (Number of reads per kilobase per million mapped reads) method for this purpose. Figure 1 illustrates the bioinformatics pipeline used for the RNA-Seq analysis. Differentially expressed genes and fold changes were derived by comparisons between stages or tissues based on DESeq and DEGSeq [7,8].

### SGR database

A relational database, the Strawberry Genomic Resources (SGR), was created to house the RNA-seq data and enable queries by users. SGR stores sample information including detailed sample description, read statistics, alignment results, gene expression raw counts, and normalized data (RPKM). Differentially expressed genes and fold changes can be retrieved by users, who specify specific pairwise comparisons.

### Web interface

**Figure 2:**
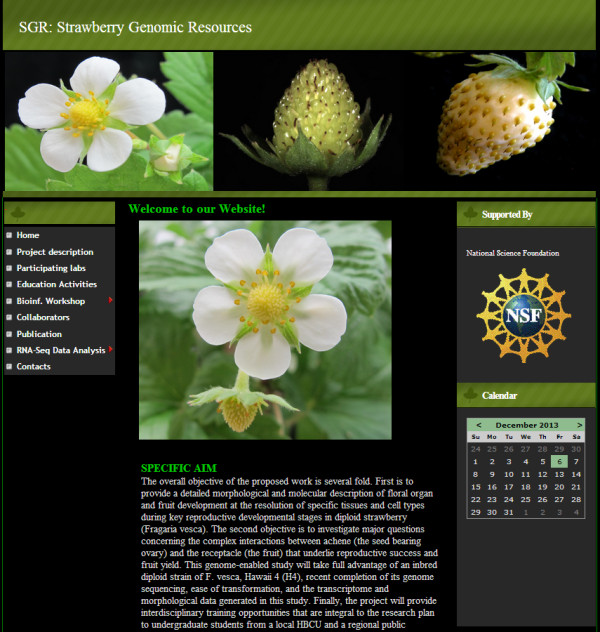
**A snapshot of the SGR home page, which describes the project and provides links to all other parts of the website.** SGR will be updated as new information is added.

**Figure 3:**
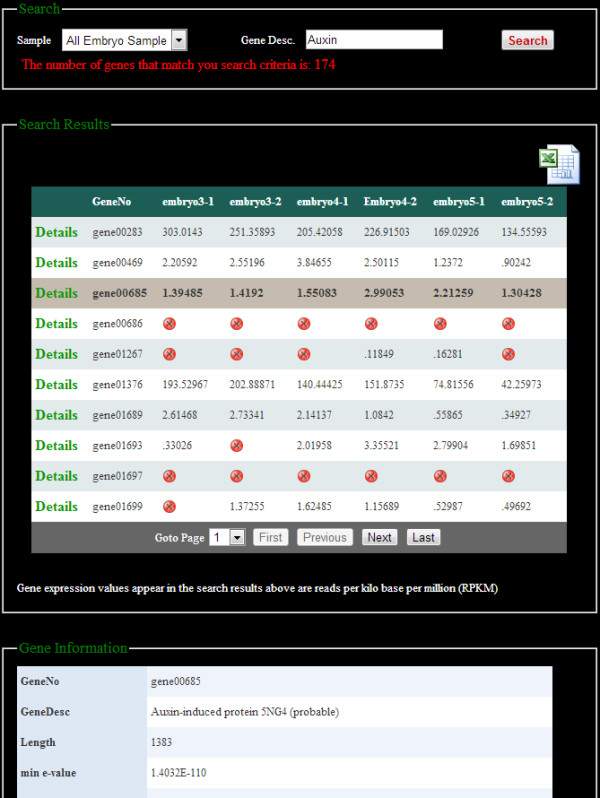
**A snapshot of a search by gene description.** Researchers can search and download gene expression profiles of the genes of interest by entering a full or partial gene description in the “Gene Desc” search box. By clicking “detail” of a specific gene highlighted in grey here, more information about the specific gene is displayed in the box below.

**Figure 4:**
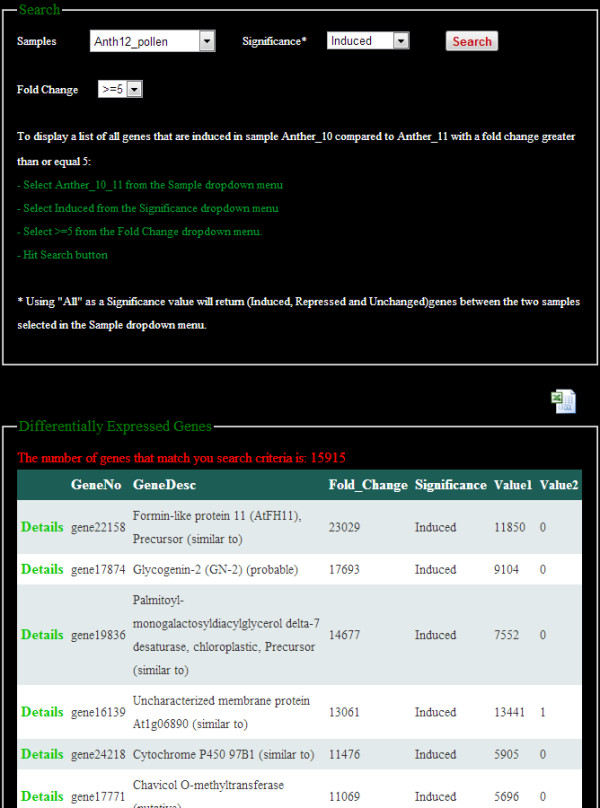
**A snapshot of the compare samples page, which allows users to view and search for quantitative differences in gene expression between tissues during development.** From the three dropdown menus, users can select a specific pairwise comparison between samples, choose to view repressed, induced, unchanged, or all genes, and specify fold change. Results can be downloaded in tabular form by clicking the “Excel” icon on the upper right.

We developed a user friendly website to allow rapid remote access to all data housed at the SGR. The website home page provides project description and has links to all the other parts of the website (Figure 2). The website provides a number of functions, such as searching, browsing and downloading the analysis results. Users can browse the gene expression data through the sample name or search for a specific gene (Figure 3). Search results can be downloaded in a tabular format at the user’s computer. The website also has the capability of searching genes by an exact or partial gene description. On the Differential Expression pages (Figure 4) users are able to select any two samples of interest for comparison and obtain differentially expressed gene names and fold changes derived with two different tools, DEGSeq and DESeq. Users are able to filter their search by selecting the significance of difference (by p-value) as well as gene expression trends: induced or repressed. Fold change can also be used as a cut off to reduce the number of genes returned.

**Figure 5:**
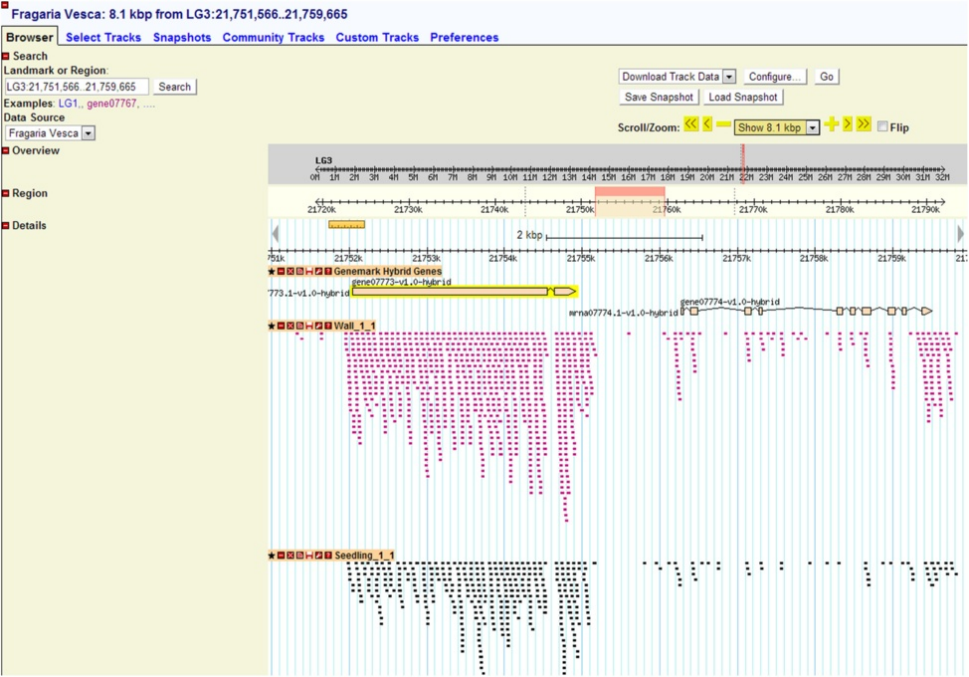
**A snapshot of the SGR GBrowse window.** Alignment of *F. vesca* transcriptomic short reads from two different tissue libraries to the *F. vesca* genome is shown along with the predicted gene models. Intron-exon gene structure, alternative splicing, and transcripts derived from non-predicted loci can be visualized.

The SGR GBrowse supports most genome browser features, including qualitative and quantitative tracks. GBrowse version 2.0 supports next generation sequencing. We deployed GBrowse to visualize the short read alignment output files against the reference genome and the hybrid GeneMark predicted genes. GBrowse allows direct insights into gene expression levels and gene structure through read alignment and read counts (Figure 5).

### Future perspectives

Transcriptome data from tissues and organs of the developing strawberry flower, including male and female organs and gametophytes, will be added to the database. These data will help scientists describe the molecular events underlying flower development in strawberry, and similar events in closely related members of the Rosaceae family such as raspberry and rose.

In addition, transcriptome data is currently being used to improve annotation of transcribed genes in the *F. vesca* genome. Current gene models are based on hybrid GeneMark predictions [1] or Gnomon (NCBI Refseq assembly GCF_000184155.1). With the vast amounts of transcriptomic data in the database we can derive more accurate gene models. Finally, we plan to identify and display SNPs between different *F. vesca* accessions, including Hawaii 4 × 4, the sequenced reference genome, and YW5AF7, a closely related accession used in the study of flower and fruit development.

## Conclusions

The SGR provides important genomic resources for scientists working with strawberry and other *Rosaceae* species, which include many important fruit crops. In addition, the database will facilitate investigations into basic questions of plant reproductive development. Together, the data, analyses, and tools will expand our ability in identifying key genes and pathways that regulate plant reproduction and crop yields.

## Availability and requirements

The database is freely available at: http://bioinformatics.towson.edu/strawberry/.

The SGR GBrowse can be freely accessed at http://mb3.towson.edu/gb2/gbrowse/F.Vesca/.

Both SGR and the SGR GBrowse can be used via any standard internet browser.

## Competing interests

The authors declare that they have no competing interests.

## Authors’ contributions

OD designed and developed the database and the user interface. SH assisted with setting up the Genome Browser. CK, CH and AG generated the RNA samples that were sequenced. JS, ZL and NA designed the research and supervised the work. OD, JS, NA, and ZL wrote the manuscript. All authors read and approved the final manuscript.
